# Coupling Capillary-Driven Microfluidics with Lateral Flow Immunoassay for Signal Enhancement

**DOI:** 10.3390/bios13080832

**Published:** 2023-08-21

**Authors:** Pooya Azizian, Jasmina Casals-Terré, Elena Guerrero-SanVicente, Ruta Grinyte, Jordi Ricart, Joan M. Cabot

**Affiliations:** 1 Energy and Engineering Department, Leitat Technological Center, 08225 Terrassa, Barcelona, Spain; pazizian@leitat.org (P.A.);; 2 Mechanical Engineering Department, Technical University of Catalonia, 08222 Terrassa, Barcelona, Spain

**Keywords:** capillary-driven microfluidics, lateral flow assay, cortisol, fluorescence spectroscopy, 3D-printing, capillary valve

## Abstract

Microfluidics has emerged as a versatile technology that is applied to enhance the performance of analytical techniques, among others. Pursuing this, we present a capillary-driven microfluidic device that improves the sensitivity of lateral flow immunoassay rapid tests thanks to offering an automated washing step. A novel multilevel microfluidic chip was 3D-printed with a photocurable black resin, sealed by an optically clear pressure-sensitive adhesive, and linked to the lateral flow strip. To depict the efficacy of microfluidics and the washing step, cortisol was measured quantitatively within the proposed device. Measuring cortisol levels is a way to capture physiological stress responses. Among biofluids, saliva is less infectious and easier to sample than others. However, higher sensitivity is demanded because the salivary cortisol concentrations are much lower than in blood. We carried out a competitive lateral flow immunoassay protocol with the difference that the microfluidic device applies an automated washing step after the sample is drained downstream. It washes the trapped quantum-dot-labeled antibodies out from nitrocellulose, diminishing background noise as these are bonded to cortisols and not to the immobilized receptors. Fluorescence spectroscopy, as a high-precision analysis, was successfully applied to determine clinically relevant salivary cortisol concentrations within a buffer quantitatively. The microfluidic design relied on a 3D valve that avoids reagent cross-contamination. This cross-contamination could make the washing buffer impure and undesirably dilute the sample. The proposed device is cost-effective, self-powered, robust, and ideal for non-expert users.

## 1. Introduction

Rapid microfluidics advancement has opened a broad avenue toward point-of-care (POC) diagnostics as one of its promising application areas [1]. Microfluidics is the science and technology of fluidic systems with micron-sized features. Miniaturization of diagnostic assays within microfluidic devices offers the benefit of using fewer reagent volumes, rapid and high-resolution analysis, cost effectiveness, and small footprints for the analytical devices [2]. Steering clear of the need for bulky and costly external instrumentation, capillary-driven microfluidics offers an on-chip fluid control approach suited for POC diagnostics [3,4]. Capillary-driven microfluidic devices control fluid flows spontaneously. This is controlled by their microchannels’ geometry and surface energy. On the other hand, lateral flow diagnostic tests rely on paper-based microfluidics, a broadly used inexpensive diagnostic substrate utilizing capillary wicking, and pre-dried reagents, which mostly provide qualitative yes/no signals for a wide range of analytes [5,6]. Recently, the combination of reagent manipulation by capillary-driven microfluidics (channel-based) and the lateral flow immunoassays presented the enzymatic amplificated quantitative POC detections [7,8,9]. For example, a quantitative measurement for severe acute respiratory syndrome coronavirus 2 (SARS-CoV-2) antibodies [7], and benzodiazepine drug abuse detection using quantum-dot-labeled antibodies [8] were reported. These new studies prospect that appending lateral flow into the preprogrammed capillary-driven chips has great potential for future POC devices.

Cortisol stands as the primary stress biomarker as it is linked with many physiological processes, such as neural development and cell death, making the diagnosis of its excessive secretion demanding [10,11]. Non-invasive and non-stressful facile sampling shortly after when the person is proximate to the influential social context makes saliva an attractive biological matrix for cortisol detection [10,12]. For healthy adults, its range in saliva was formerly reported to be between 10.2 and 27.3 ng/mL in the morning and 2.2 and 4.1 ng/mL at night [13,14]. The cortisol detection using direct sandwich immunoassay is challenging due to its low molecular mass (362.46 Da) [11,12]. It does not provide multiple binding sites [12]. However, it can be measured using a less sensitive competitive immunoassay (compared with sandwich immunoassay) [11,15]. Even so, the quantitative measurement using competitive assay mostly has a narrow detectable range, which is more evident in calorimetry, typically with a concentration detection limit of approximately 100 ng/mL [16]. This limit disrupts salivary quantitative cortisol sensing, considering its low concentration range. Therefore, further studies addressing the detection of low-concentration analytes have been done to enhance the detection limits to about 1 ng/mL using methods like preincubation, trapping not bonded gold nanoparticles, aptamer-based bioassay, and strengthening the detection setup [11,12,16,17]. Another approach is the use of conjugating fluorescent labels, which offer higher sensitivity than most biomolecules [18]. Among fluorescent labels, quantum dots (QDs) are the most promising choice which improves detection limits due to their intrinsic properties like high yields, lifetime, and stability [19]. Using QDs conjugated to antibodies showed great capability while employed in other sensitive detections, like for drugs, decreasing the detection limit by approximately two orders of magnitude [19,20]. However, the trapped QDs in a lateral flow test can provide background noise in the entire strip, which we aimed to address here by appending a capillary-driven microfluidic device into the test.

Here, we proposed to combine new 3D capillary-driven microfluidics and a lateral flow strip to enhance the current operational concentration range for the fluorescent lateral flow test. In addition, quantum-dot-labeled antibodies were employed for quantitative salivary cortisol detection. The microfluidic device introduces an automated washing step, using a novel 3D capillary valve to avoid reagent cross-contamination and dilution. We studied the effect of this appended step on the cortisol immunoassay, showing the advantage of using microfluidics combined with the lateral flow assays.

## 2. Materials and Methods

### 2.1. Immunoassay Protocol

The employed competitive assay forms a measurable emission at the test area in the absence of cortisol in the sample. This occurs where the remaining free anti-cortisol antibodies, available in the sample buffer, bond to the immobilized cortisol-BSA (the conjugates of cortisols to a heavier protein: bovine serum albumin). These conjugates were pre-immobilized in the detection area (on the nitrocellulose) as the receptors. Since the anti-cortisol antibodies are labeled by fluorescent nanoparticles, the test area is detectable using fluorescence spectroscopy, and the cortisol concentration in the sample is inversely proportional to the fluorescent light intensity there. So, the immunoassay protocol respectively comprises (i) adding the sample into the buffer of anti-cortisol antibodies, where cortisol/anti-cortisol interactions may occur; (ii) introducing the buffer into the microfluidics, which subsequently flows through the lateral flow strip; (iii) forming the bonds between the free anti-cortisol antibodies and the immobilized cortisol-BSA; and (iv) washing and removing the antibodies that attached to the cortisol from the sample and not to the immobilized conjugated ones in the detection area.

### 2.2. Solution Preparation

The washing solution was phosphate-buffered saline (PBS) containing 0.05% (*v*/*v*) Tween^®^ 20, pH 7.4 (Merck, Manama, Bahrain). For sample preparation, 10 µL of sample solution containing different concentrations of cortisol (0–2000 ng/mL) in PBS was mixed with 10 µL of 250 µg/mL anti-cortisol antibodies and 1:100 secondary antibodies F(ab’)2-Goat anti-Mouse IgG (H + L) conjugated with quantum dots (Qdot™ 605, Thermo Fisher Scientific, Waltham, MA, USA) in 2% (*w*/*v*) bovine serum albumin (BSA) with 1% (*v*/*v*) Tween^®^ 20. Therefore, the final solution concentrations were 0–1000 ng/mL cortisol, 125 µg/mL Anti-cortisol, and 1:200 QD-Ab within a PBS Tween (0.5%) BSA (1%) buffer. Herein, Tween and BSA represented and functioned as surfactants, which respectively offer a decrease in the surface tension to secure a steady lateral flow through the nitrocellulose and avoid non-specific protein binding.

### 2.3. Microfluidic Device

**Figure 1:**
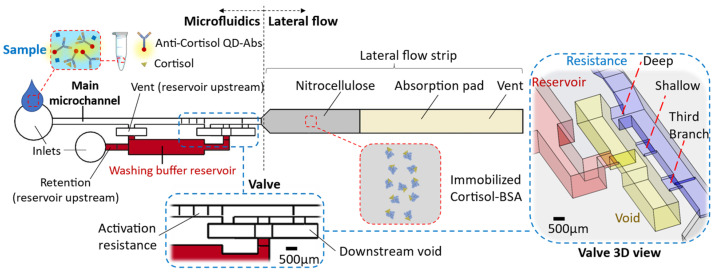
The chip schematic encompasses a microfluidic device and lateral flow strip. The microfluidic comprises a main microchannel that is connected to the washing buffer by way of a valve.

The chip comprises two main parts: (i) the microfluidic device and (ii) the lateral flow strip located at the microfluidics downstream (Figure 1). The microfluidic device encompasses the main microchannel (width: 700 µm × height: 500 µm) and the washing buffer reservoir (20 µL). There are inlets to introduce the washing buffer within the reservoir and the sample through the main microchannel. The reservoir section is connected to the main microchannel and forms a circuit by way of a valve at its downstream (Figure 1). The valve specifications and their reasoning are described in the results. Impacts, like when a chip falls, can release the washing buffer from the capillary valve. To avoid such a valve failure, we consider a relatively high retention upstream of the reservoir to hold the buffer (≈1500 Pa, height: 500 µm × width: 50 µm, for the inlet microchannel into the reservoir). Considering this high retention and the resistance of the circuit up to the absorbent pad, in order to release liquid from the reservoir, an upstream venting for the washing buffer reservoir is required. This venting (700 µm × 500 µm) is connected to the main microchannel that lets air displace into the reservoir only when the sample is emptied from there, while from the side of the downstream valve, enough suction is provided.

The microfluidic device was 3D printed using a stereolithography (SLA) printer (Form 3, FormLabs^®^, Somerville, MA, USA) with clear or black photocurable polymer (V4 resin) depending on the detection purpose. We printed the microfluidics with an open side to avoid trapping the resin during the printing process and improve the possibility of optical detection. The resin surface was activated by vacuum air plasma and coated using acrylic acid fume to make the surface hydrophilic (water contact angle: ϴ = 30° ± 5°). A series of contact angle measurements with time assured the maintenance of the desired hydrophilicity for more than two weeks. Then, a pressure-sensitive adhesive (ARclear 93495, Adhesives Research^®^, Limerick, Ireland—a 1.6 mm thick optically clear silicon adhesive layer—ϴ ≈ 90°) was used to seal the microfluidic device.

### 2.4. Lateral Flow Strip

The lateral flow strip (3 mm wide) contains a nitrocellulose membrane and an absorbent pad. The strip front, built of nitrocellulose (Cytiva^®^, Marlborough, MA, USA, Whatman™ FF80HP membrane obtained from Thermo Fisher Scientific), is placed within the end opposite to the microfluidic inlet. At 1 cm down from the strip front, the nitrocellulose connects to an absorbent pad made of multiple polyester/cellulose paper layers (VWR^®^, Radnor, PA, USA, Spec-Wipe^®^ 3). A drop of 0.5 µL cortisol-BSA 500 µg/mL solution is drop-casted in the middle of the nitrocellulose and left for 15 minutes to dry up at room temperature before integrating the lateral flow section within the microfluidics end. The lateral flow strip end is vented so air can be displaced out.

### 2.5. Detection Setup

The Dino-Lite digital microscope was used for optical and fluorescent detections. For fluorescence spectroscopy, 405 nm excitation and 610 nm emission wavelengths were used. For this, a colored glass filter (FGL610S, 610 nm long-pass, Thorlabs, Inc., Newton, MA, USA) was utilized to sort out other wavelengths except for the quantum dots (QDs) emissions. Exposure time was set at 1/8 s (except for the case that it is specified), and power and gain were kept the same in all fluorescent detections. The detection was carried out inside a black box and using 3DP black resin microfluidic chips to reduce the light noise. Clear resin 3DP parts and deionized water (mixed with 10% *v/v* food dyes) were utilized for illustration purposes.

## 3. Results and Discussion

A new capillary-driven microfluidic device was designed and used to append a subsequent washing step for lateral flow assay. It was tested for quantitative cortisol detection as a proof of concept. The fluorescence measurements were done using quantum dots (QDs) labeled antibodies to enhance the sensitivity of the typical qualitative lateral flow assay to a quantitative clinically relevant salivary range. Appending the washing step is more impactful in the competitive immunoassays, for instance, avoiding the QDs’ fluorescence background noise.

### 3.1. Washing Step Effect

**Figure 2:**
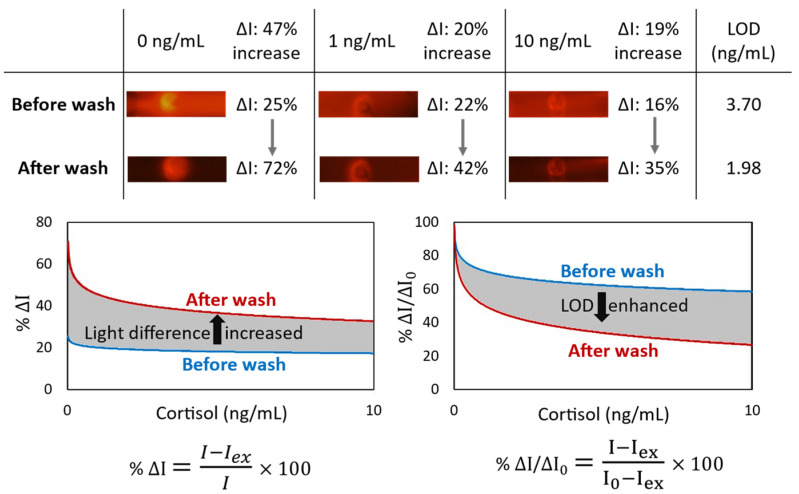
The effect of appending an automated washing step by the microfluidic device on the lateral flow immunoassay of cortisol. For the blank sample, the light intensity difference (ΔI) between the targeted detection circle and the rest of the nitrocellulose is increased by 47%. Also, LOD (by SD_0_ from 3 replicates) is enhanced by 1.87 times.

In a competitive immunoassay, the signal intensity is inversely proportional to the antigen concentration. In other words, competitive immunoassays limit the number of antigen binding sites, forcing a target analyte and a labeled ana-log to compete for antibody binding. Given that the unlabeled analyte has a higher affinity for the antibodies than the labeled analog, the quantity of bound labeled analyte is inversely related to the concentration of the analyte of interest. For the competitive cortisol immunoassay, the cortisol-BSA molecules were immobilized on nitrocellulose, which was used to capture the free anti-cortisol antibodies from the sample. Conjugation of cortisol with proportionally larger and heavier molecules such as BSA helps for better immobilization. While the utilized antibodies are fluorescent conjugated, the cortisol concentration in a sample is inversely proportional to the fluorescent light intensity at the detection area of the lateral flow. Although immobilized cortisol-BSA is only available at the specific section of the nitrocellulose to capture the antibodies, some antibodies were physically trapped through the membrane, providing background noise. It could happen even for the antibodies bonded to the cortisol molecules, especially when antibodies are conjugated to the proportionally larger and heavier fluorescent nanoparticle. We observed that the sensitivity of a lateral flow immunoassay could be improved by appending a subsequent automated washing step to remove these trapped antibodies from the nitrocellulose (Figure 2). The light intensity difference (ΔI) between the targeted detection area and the rest of the nitrocellulose is increased by 47% for the blank samples. Moreover, the limit of detection (LOD) is enhanced by 1.87 times.

### 3.2. Valve Effect

**Figure 3:**
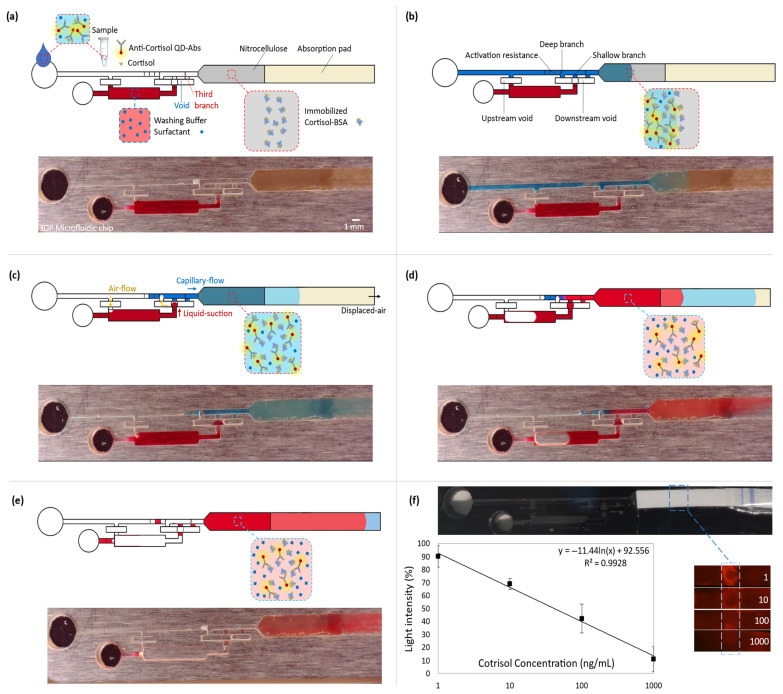
Coupling the microfluidic device with the competitive lateral flow immunoassay for quantitative cortisol measurement. All the top rows (**a**–**e**) represent the schematics of the immunoassay steps, and the bottom rows show the capillary-driven flow experiment at the corresponding immunoassay stage. To have a clear vision of the fluid dynamic steps, the colored aqueous phases were used in a transparent microfluidic chip. While fluorescent spectrometry was done within the 3D-printed black chips. (**a**) Introducing the sample into the capillary-driven microfluidic device. (**b**) Capillary action drives the flow from the inlet into the downstream lateral flow, which contains immobilized cortisol-BSA on nitrocellulose. Free antibodies bond to the cortisol-BSAs. (**c**) The sample (blue) flows downstream, and the suction of the absorption pad empties the main microchannel until the shallow resistor before the valve junction. It opens a venting channel, upstream of the washing reservoir. Then, the downstream suction displaces air from the void into the deep branch. It moves the washing buffer (red) into the void and connects it to the blue liquid through the second branch. (**d**) The valve is opened, and the washing buffer goes through the lateral flow part. (**e**) The washing step is finished, which removes the free QD-Ab from the nitrocellulose. It increases the fluorescent light intensity difference between the detection point and the nitrocellulose background. (**f**) The obtained calibration curve for cortisol. The 3D-printed chip is black to decrease surrounding noise. By increasing the cortisol concentration in the sample, fewer free QD-Abs are available to bond to the receptors. So, cortisol concentration shows a reverse relation with the fluorescent light intensity (*y* = −11.44 *ln*⁡(*x*) + 92.556). The maximum and minimum reference lights are for blank and 1000 ng/mL samples, respectively (SD from 3 replicates).

In capillary-driven flows, sudden expansions in the cross-section of microchannels change the meniscus curvature of the filling front. It locally decreases the magnitude of capillary pressure. Therefore, an abrupt enlargement in a microchannel can eliminate the driving net force and stop the flow [3,4]. For instance, a capillary flow within a smaller microchannel stops at the junction with a bigger microchannel. Afterward, a flow in the bigger microchannel can trigger and re-activate the stopped flow by breaking the stopped liquid–air meniscus at the junction. To program a flow sequence, in which first the triggering flow thoroughly drains, then the stopped liquid flows, the latter should be kept by an extra retention force until the demand. A retention pressure from upstream can apply this force. For instance, an upstream constriction (burst valve) retains the liquid until a downstream suction overcomes it. In our device, this retention is applied by an alternative venting connected to the main microchannel. Therefore, the reservoir venting opens after the triggering liquid is emptied from the main microchannel, securing a successive release. At a trigger junction, after breaking the liquid–air meniscus until compelling the upstream retention and release, diffusive mixing of fluids’ molecules can lead to sample dilution, washing buffer contamination, and experiencing non-lateral flow patterns downstream [8]. A mediator gap filled by air can isolate the liquids to avoid these issues [4]. However, utilizing an air gap can later result in bubbles entering the circuit and microfluidic malfunction [8]. Therefore, a deliberate valve configuration is required to displace the air and avoid introducing bubbles (Figure 1); this workflow is discussed in the next section and illustrated in Figure 3a–e.

In summary, the utilized valve offers two distinctions: (i) the reagents are isolated by a gap instead of contacting at a trigger junction, and (ii) a complete liquid drain from the main channel opens an upstream venting for the reservoir. Therefore, the release is not based on a retention burst.

### 3.3. Microfluidics Function

To enhance the immunoassay signal, the microfluidic device offers an automated washing step after introducing the sample into the chip. The washing buffer is kept within its reservoir by a valve and is designed to be released after draining the sample. To retain the buffer, an abrupt 3D enlargement (void) stops the capillary-driven flow from leaving the reservoir (Figure 3a). This is achieved by a decrease in the magnitude of the local capillary pressure. Moreover, an opposing capillary pressure from the sudden upstream constriction at the reservoir entrance secures keeping the liquid. In a trigger valve, the stop junction forms by expanding into the trigger channel (the main microchannel). However, in the diffusion-free valve, the expanded junction is embedded in the void. The void functions as an intermediary between the main microchannel and the reservoir. When the sample is introduced into the main microchannel, a trapped air gap within the void isolates the reagents. Therefore, it avoids possible convection or even diffusion of the reagents into each other (Figure 3b). Three branches connect this void to the main microchannel (all the widths are the same: 700 µm). From the upstream, the first one is the deepest (500 µm), which has the least capillary retention and resistance compared with the rest of the branches. It functions for valve activation. The second one is shallower than the first (100 µm), operating for the liquid connection. Finally, the third branch, which has the same dimensions as the second branch, works to vent and displace the air out from the void. It lets the liquid thoroughly fills the two previous branches. The absorbent pad displaces the sample (colored blue in Figure 3) from the main microchannel into the lateral flow section. It opens the upstream vent of the reservoir. There is a resistor within the main microchannel and before the valve junction (a decrease in the microchannel depth from 500 µm to 100 µm and with a 3 mm total length). Once the sample is emptied up to there, the activation resistance holds the liquid by its high capillary retention. Since this retention is higher than the equivalent retention of the valve and the reservoir, the downstream suction power displaces the air from the void into the deep branch. This air displacement is essential to prevent an air bubble from entering the shallow branch and, subsequently, the downstream circuit. Air bubbles can lead to flow malfunction and failure of the device. This pneumatic suction pulls the washing buffer (in red) into the void (Figure 3c). The washing buffer joins to the flow-end through the shallow branch. As a result, the valve opens, and the buffer flows into the lateral flow strip (Figure 3d). Later, the absorbent pad empties the washing buffer. Therefore, it provides an automated washing step after the lateral flow assay through the nitrocellulose (Figure 3e).

### 3.4. Quantitative Cortisol Detection

Quantitative cortisol detection was performed by a competitive immunoassay based on the interaction of immobilized cortisol-BSA molecules and free anti-cortisol antibodies. The experimental results of the assays appending the washing step are reported in Figure 3f. The calibration curve shows that the mean of light intensity decreases by approximately 80 percent at the detection section on the nitrocellulose for the increase in the cortisol concentration from 1 ng/mL to 1000 ng/mL in the introduced samples into the chips. With lower concentrations of cortisol in the sample, more antibodies are available to attach to the immobilized cortisol-BSA molecules, leading to higher light intensity. The regression equation of *y* = −11.44 *ln*⁡(*x*) + 92.56 represents the averages of the scanned data (3 replicates per concentration). It shows that QD-Ab has a high sensitivity for cortisol detection with a 2.0 ng/mL limit of detection (LOD = exp((3 × SD_0_)/slope)) for our blank assay, which indicates a better performance than the traditional cortisol immunoassays (LOD ≈ 100 ng/mL) [16], using colloidal gold nanoparticles mostly for qualitative detection. Also, considering the previous studies that utilized QD-Ab for fluorescent detection (LOD ≈ 5 ng/mL) [19], the appended washing step improved sensitivity and LOD more than twice by decreasing the background noise. Video S1 shows the cortisol competitive immunoassay for a blank sample (20 times faster) while using an exposure time of 1/2 s. It should be mentioned that there are other strategies to optimize sensitivity, such as the optimization of reagents, which are out of the scope of this paper.

## 4. Conclusions

A versatile diagnostic device was designed and fabricated based on the combination of multilevel microfluidics and lateral flow assay. The device was proved to be compatible with competitive immunoassays. Appending an automatic washing step increases the detection sensitivity by improving the light intensity difference between the detection area and its background by about 47% for the blank sample. We successfully applied fluorescence spectroscopy of the different cortisol concentrations (1–1000 ng/mL) within the microfluidic device, showing a detection limit of 2.0 ng/mL. Embedding the washing step instead of using separated components like vials improves the cost effectiveness of lateral flow assays requiring further wash. However, for mass production, fabrication methods like injection molding should be considered instead of prototyping by 3D printing. In summary, this work demonstrates a cost-effective, versatile, self-powered, and user-friendly approach for optimizing immunoassay tests by an automated microfluidic wash, proven with the quantification of cortisol in spike samples.

## 5. Patent

P.A., J.M.C., J.R., and J.C. are joint inventors on the patent application PCT/ES2021/070720, which covers the utilized capillary valve concept (international publication number WO 2023/057659 A1).

## Data Availability

All data generated or analyzed during this study are included in this published article (and its Supplementary Information Files) or are available from the corresponding author upon reasonable request.

## Supplementary Materials

The following supporting information can be downloaded at https://www.mdpi.com/article/10.3390/bios13080832/s1. Video S1: the cortisol competitive immunoassay by appending the microfluidic device to the lateral flow strip (blank sample, 20× faster, exposure time of 1/2 s).
